# Polyether-based polyurethane electrolyte for lithium metal battery: a perspective

**DOI:** 10.1039/d4ra06863g

**Published:** 2024-11-11

**Authors:** Peng Cui, Yifan Li, YuXing Liu, Siqi Wang, Xingyu Tang, Yihong Ye, Hance Su, Chun Sun

**Affiliations:** a School of Mathematical and Physical Sciences, Nanjing Tech University China cuipeng1413@njtech.edu.cn; b School of Engineering Auditing, Nanjing Audit University China 270330@nau.edu.cn; c Department of Materials Science and Engineering, Standford University USA yuxingl9@stanford.edu hancesu@stanford.edu; d Nanjing Pinnacle New Energy Ltd China 18018030732@163.com; e Department of Chemistry and Biochemistry, University of California Los Angeles USA yeyihong@ucla.edu chunsun1@ucla.edu

## Abstract

Polyurethane (PU)-based electrolyte has become one of the most important research directions because of its unique repeating ‘soft–hard’ segment co-polymer structure. Its ‘soft segment’ composition includes polyethylene oxide, polysiloxane, polycarbonate, cellulose and polyether. Among them, polyether-based polyurethane electrolytes (PPES) have the advantages of simple synthesis, molecular structure optimization and functional group modification, which can greatly improve the ionic conductivity of the system and form a good ion transport interface. To date, a few separate and detailed reviews of advances in PPES have been reported. In this paper, the research progress of PPES is reviewed from the aspects of structural design strategy, molecular synthesis, conductivity modification methods, specific functions and interfacial ion transport behavior in lithium metal batteries (LMBs). In addition, the synthetic route of PPES and the development prospect of PPES are discussed. We also provide guidance for developing high-performance PPES for next-generation LMBs.

## Abstract 2

Mechanism of increasing ionic conductivity of polyether-based polyurethane electrolyte.

## Graphical abstract

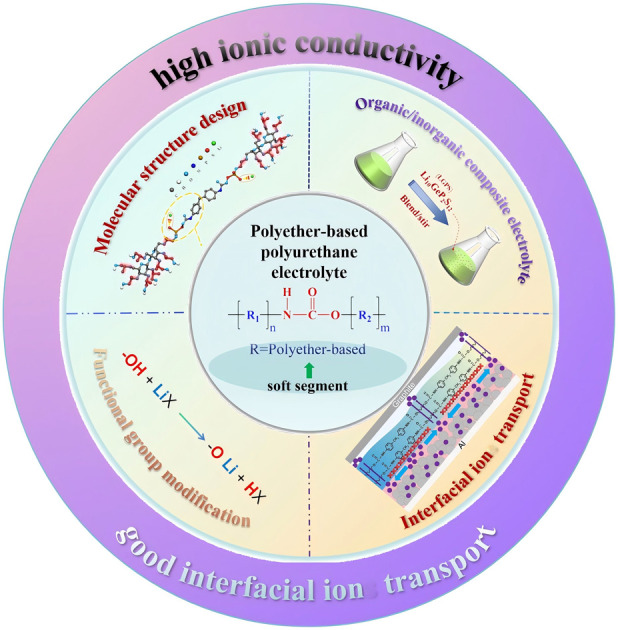

## 1 Introduction

All-solid-state lithium metal batteries (ASSLBs) have become one of the key directions of energy storage devices because of their high energy density, high safety and excellent cycling life.^1^ As a core component of the battery, solid electrolyte works as an electrode separator, ion conductor and dendrite inhibitor. Solid polymer electrolytes (SPEs) have become the focus of the industrialization of all-solid-state batteries because of their excellent flexibility and molecular tailoring direction in comparison with inorganic electrolytes, such as ceramic oxides/sulfides.^2^

At present, the main research subjects of polymer electrolytes are polyethylene oxide (PEO),^3^ polycarbonate (PC),^4^ polyurethane (PU) and so on.^5^ PEO is the earliest discovered and most widely studied type of polyether electrolyte; however, its very low intrinsic conductivity (<10^−5^ S cm^−1^) results in poor rate performance. In addition, the poor mechanical properties of PEO cannot effectively inhibit lithium dendrites, resulting in short-circuit risk.^6,7^ Polycarbonate (PC) has the characteristics of rotatable flexible molecular chains, which have attracted extensive attention from researchers.^8^ However, in the process of lithium deposition/dissolution with high current and high surface capacity in this system, a large number of uneven strips of lithium are produced, which affects the performance of battery devices.

As a kind of polymer electrolyte, PU-based electrolytes have attracted extensive attention because of their unique repeating ‘soft–hard’ segment structure, which has the advantages of flexibility, good ion transport ability and good compatibility with other polymer substrates and inorganic fillers.^9^ The typical repeating unit in PU is an amino group (–NHCOO–), which is produced by the reaction between the isocyanate (–NCO) and the polyol. The ‘soft segment’ is mainly composed of polyether, polyester and other structures, which can not only have a strong dissolution capacity for lithium salt but also be conducive to the transportation of Li^+^.^10^ The ‘hard segment’ is mainly formed by the reaction of isocyanate, diamine, *etc.*, which ensures mechanical strength.^11^ In addition, the hydrogen bond formed by ‘–NH’ on the PU molecular chain can promote the interaction between PU and inorganic fillers, and the polar functional group can accelerate the dissociation of lithium salt, thereby improving the ion transport capacity and enhancing the electrochemical window.^12^ Moreover, the hydrogen bond interaction of PU gives it good self-healing and excellent bonding characteristics,^13^ ensuring that PU has excellent interphase compatibility and safety performance. More importantly, PU molecules with specific properties can be obtained by modifying the molecular structures, such as functional group modification and side chain modification. Therefore, fully carrying out the research and development of PU-based electrolytes will be of great significance for the development of high-performance polymer-based electrolytes.

Ionic conductivity is one of the important indicators to evaluate the performance of electrolytes. The ion transport in PU-based electrolytes mainly depends on the ‘soft segment’ structure of molecules,^9^ so it is particularly important to focus on the design and development of PU molecular ‘soft segment’ structure. At present, the ‘soft segment’ structures of PU-based electrolytes mainly include polyester/polycarbonate, polysiloxane, cellulose and polyether.

Among them, polysiloxane-based PU electrolytes usually exhibit low glass transition temperature (*T*_g_) ∼ (−123 °C) and high stability. However, its poor dissolution of lithium salt limits the improvement of ionic conductivity. For example, in Ren's paper,^14^ the synthesized polysiloxane-based PU electrolyte yields a Li^+^ transfer number ∼0.80. Although the enhanced segment mobility of flexible “Si–O” units leads to a low *T*_g_, the ionic conductivity of that electrolyte is 3.7 × 10^−5^ S cm^−1^ and the performance of the assembled batteries is far from satisfactory. Polycarbonate-based PU electrolytes possess a high mechanical strength but low ionic conductivity and poor flexibility. Although the ionic conductivity of PU-based electrolyte with polyester/polycarbonate as a ‘soft segment’ can reach 1.58 × 10^−3^ S cm^−1^ (60 °C),^15^ the ionic conductivity and battery cycle performance at room temperature (25 °C) are not particularly outstanding. Cellulose is a natural polymer material with excellent mechanical strength and it can serve as a potential ion channel due to its tendency to form ordered microstructures in specific directions.^16^ However, the ionic conductivity of PU-based electrolytes with cellulose as the ‘soft segment’ is very low (8 × 10^−5^ S cm^−1^, 30 °C), which is not suitable for polymer electrolytes. In comparison, polyether-based PU electrolytes (PPES) provide lower *T*_g_ and a good dissolution site for lithium salts due to the interaction between ether bond ‘–O–’ and Li^+^ [deforming/forming lithium–oxygen(‘Li–O’) bonds]. Besides, PPES has attracted more and more attention because of its rich multipolar functional groups and easy molecular modification.^17^ Its synthesis involves not only a simple process but also high ionic conductivity at room temperature (10^−3^–10^−4^ S cm^−1^).^5,10^ Therefore, using polyether as the ‘soft segment’ structure of PPES is the promising solution to the development of high-performance PU-based electrolyte.

**Fig. 1:**
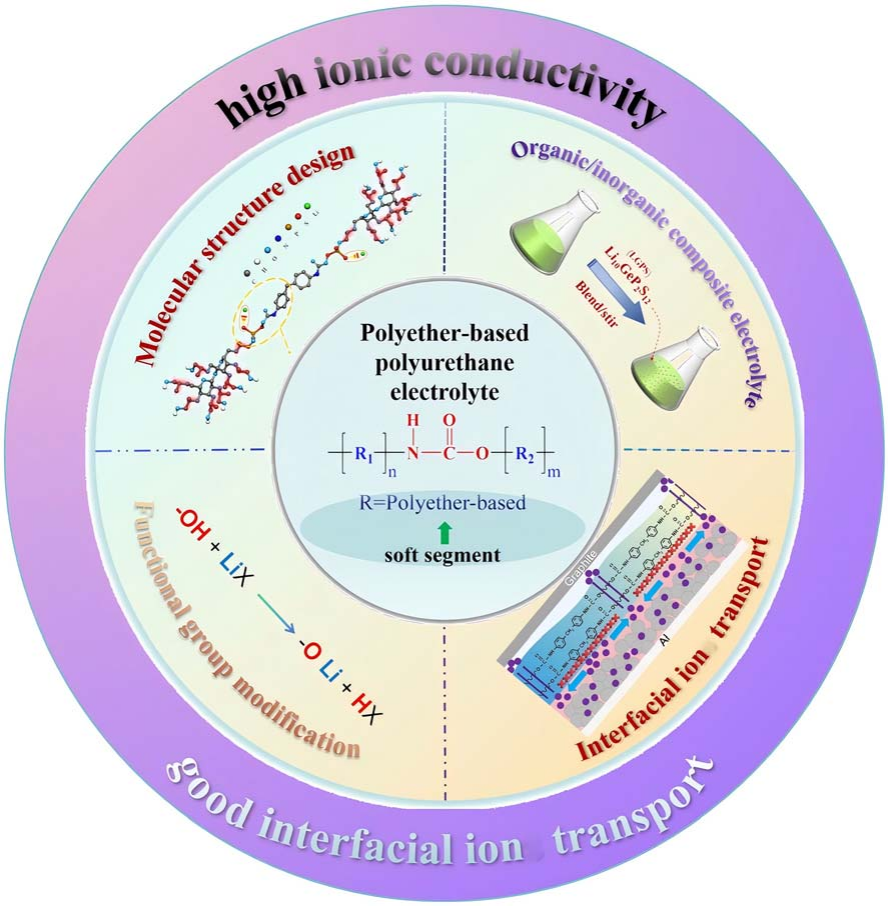
Molecular structural designs, typical modification methods, specific functional applications and advantages of PPES.

In this review, we summarize the main research progress of PPES from the aspects of ion-transport mechanism, ionic conductivity enhancing methodology, stability and interfacial ionic transition resistance (Fig. 1). Additionally, we also focus on the molecular modification and filter interaction of PU-based electrolytes aiming to optimize the ionic transport, interfacial resistance and mechanical properties. We hope this review will provide clear guidance and additional insights into the issues facing PU-based electrolytes, facilitating the exploration of PU-based electrolytes towards practical applications.

## 2 Mechanism of ion transport of PPES

Ionic conductivity is an important indicator to measure the performance of SPEs, and its level is related to whether the electrolyte can be directly applied to the batteries. Therefore, the in-depth study of the ion conduction mechanism in solid polymers can provide an important theoretical basis for the design and preparation of efficient SPEs in the future. The traditional ionic conductivity is calculated using the following formula:^18^

1 *σ* = ∑*c*_i_·*q*_i_·*μ*_i_

In the formula, *σ*, *c*_i_, *q*_i_ and *μ*_i_ represent ionic conductivity, ionic charge, carrier concentration and carrier mobility, respectively. According to the formula, the way to improve the ionic conductivity is to increase the carrier mobility and concentration. Generally, the carrier concentration (number) is closely related to the dielectric constant of the polymer because a high dielectric constant can improve the solubility of lithium salt. Many relevant references show that in most solid polymer electrolytes (SPE), ion transport mainly depends on amorphous (amorphous) regions. Carrier mobility is closely related to the polymer glass transition temperature (*T*_g_). Therefore, polymers with low *T*_g_ (<0 °C) tend to have higher ionic conductivity. The level of carrier concentration also has an important impact on the level of ionic conductivity. The relationship between *σ* and *q*_i_ is mainly reflected in two aspects:^19^ (1) in the polymer system, Li^+^ can be regarded as charge carriers on the premise that low-concentration lithium salts are completely integrated into the polymer system, and the amount of lithium salts added can be calculated according to the charge carriers. However, when the high concentration of lithium salt cannot be completely dissolved, the electrostatic interaction between the ions will affect the ionic conductivity. In this case, the ionic conductivity of the system may decline and cannot be calculated by the carrier formula. (2) In the integration of lithium salts into the system, there are interactions between lithium salts and polymers, including ion/dipole interaction, the adsorption of lithium ions on various polar functional groups in the polymer system, which will affect the ionic conductivity of polymer electrolytes. The ion transport mechanism of polymer electrolytes will be explained step by step according to different theoretical models and equations in the following parts.

### 2.1 Theoretical model of ion conduction in polymers

#### 2.1.1 Crystal vacancy diffusion model and non-crystalline zone diffusion model^20^

**Fig. 2:**
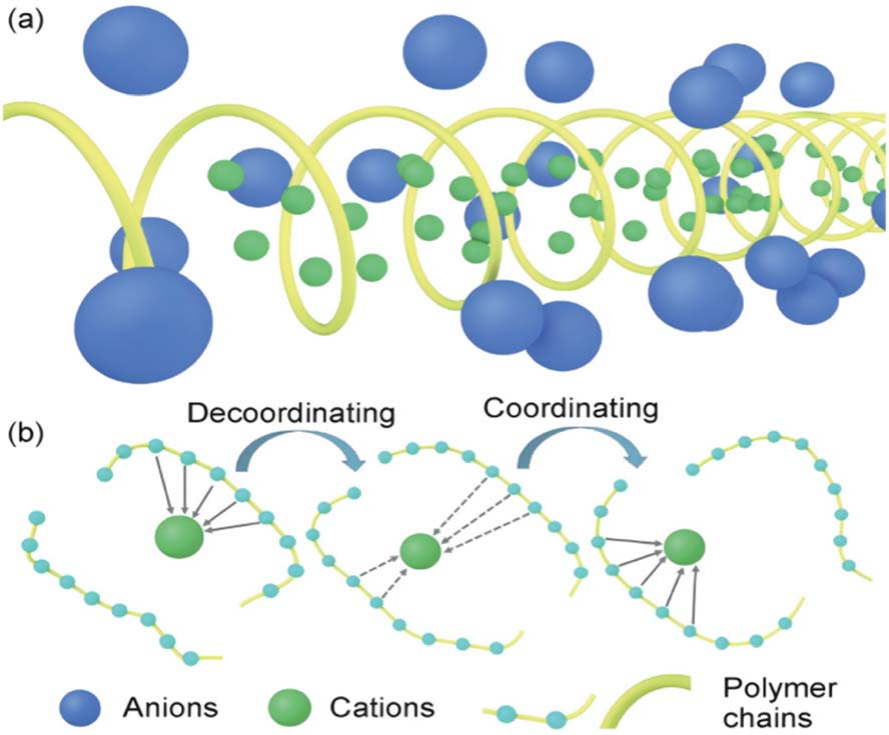
Conduction mechanism of the solid polymer electrolyte. (a) Crystal vacancy diffusion model; (b) diffusion model in amorphous region.^21^

The crystal vacancy diffusion model is shown in Fig. 2(a), which mainly studies the conduction process of ions in the crystalline and non-crystalline regions of the polymer. The polymer in the crystalline region mainly presents a spiral shape. Under the action of an applied electric field, lithium salts dissociate into anions and cations. The cations are conducted inside the spiral pipe, while the anions are conducted in reverse outside the pipe. The helical conformation of different polymers may be different, but the canal-cation interaction exists all the time and affects the ionic conductivity of the polymers. Currently, researchers have studied PEO polymer electrolytes the most. In 1982, Shriver *et al.* found that cations were conducted through helical orbits in PEO,^22^ confirming the PEO conduction mechanism proposed by Armand in 1979.^23^ In 1999, Bruce *et al.* proposed that cations and anions conduct conduction inside and outside the pipeline, respectively.^24^ In 2003, this result was further proved by theoretical calculations and other characterization methods.^25^

Although the crystalline zone model explains part of the conduction mechanism to a certain extent, most polymers are in semi-crystalline or amorphous states at room temperature. This phenomenon leads to that the ion conduction is mainly carried out in the amorphous zone, thus deriving the ion conduction model in the amorphous zone.

As shown in Fig. 2(b), in the non-crystalline zone conduction model, the polar functional groups in the polymer system can form Lewis acid–base pairing with lithium ions, thereby improving the degree of dissociation of lithium salts. Then, the “ligation–dissociation–coordination” process continues with the polymer connection movement to achieve ion conduction. Currently, many researchers have studied the conduction of PEO polymers in the amorphous zone to improve the electrolyte properties.^26^ According to the current conduction mechanism of polymer in the amorphous region, the ionic conductivity depends mainly on the kinematic ability of the chain segment in the high elastic region. Increasing the amorphous region of the polymer by copolymerization, blending, crosslinking and adding fillers have become the main methods to improve the ionic conductivity of polymer electrolytes. XIA^26^ used cryo-electron microscopy, deep X-ray photoelectron spectroscopy and atomic force microscopy to study the composition and mechanical properties of the amorphous regions of ethers and esters. Based on this, we propose that the coulomb efficiency of lithium deposition in the ester electrolyte is as high as 98.5% by optimizing the proportion of the amorphous region. Excellent cycle performance is shown in both finite lithium metal (N/P = 0.5, 1) and lithium-free full batteries.

#### 2.1.2 Free volume model and Vogel–Tamman–Fulcher (VTF) equation

The diffusion of ions in a polymer system is affected by many factors, which are further explained by the free volume model. This model points out that the polymer chain movement occurs when the polymer conducts ions, and the volume of the space around the molecule changes with the chain movement, which is called the free volume. The size of the free volume is directly proportional to the movement energy of the polymer chain. When the free volume space is smaller than the volume of ions produced during the dissociation of lithium salt, the temperature is below the polymer *T*_g_, which is equivalent to almost no movement of the molecular chain and no ion conduction process. When the free volume space is greater than the volume of ions produced during lithium salt dissociation, the temperature is above the polymer *T*_g_, the polymer molecular chain movement is enhanced, and the ions will produce a conduction process. Based on the free volume model, the researchers used the Vogel–Tamman–Fulcher (VTF) equation to arrive at a conclusion:2
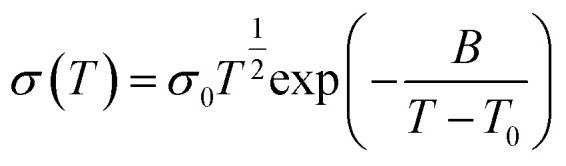
In this formula, *T*_0_ and *T* represent the glass conversion temperature, ionic conductivity of the apparent activation energy of the ambient temperature, respectively. According to the VTF equation, the lower the *T*_g_ in the polymer electrolyte, the higher the ionic conductivity.^27^ Therefore, in previous studies, researchers mainly focused on improving the conductivity of the electrolyte *T*_g_ to improve the ionic conductivity.^20^ Although some results have been achieved, low *T*_g_ results in a decrease of the mechanical strength of the polymer electrolyte,^28^ resulting in dangers such as short circuits.^29^ Therefore, it is still challenging to develop polymer electrolytes with both good mechanical strength and ionic conductivity.

## 3 Molecular design strategy for preparing PPES with high ionic conductivity

### 3.1 Preparation of electrolytes with high ionic conductivity by molecular structure

**Fig. 3:**
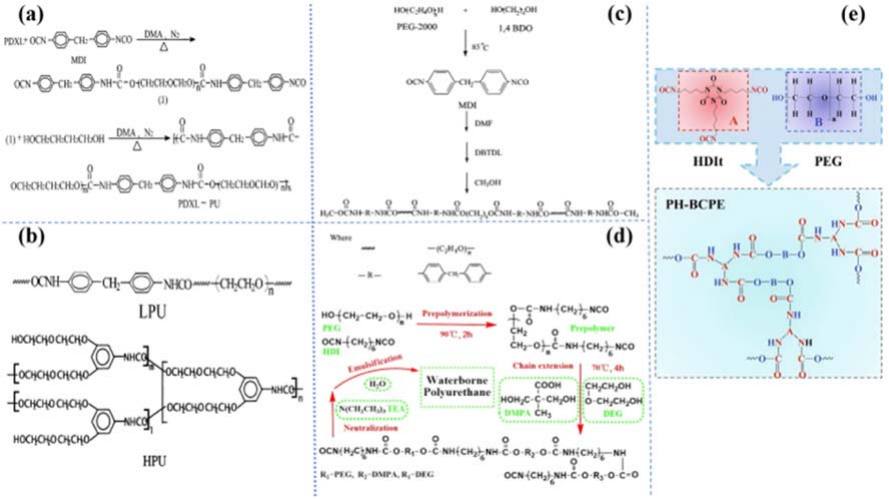
(a) Synthesis of PDXL-PU; (b) polymerization of the TPUs; (c) synthesis of LPU and HPU; (d) synthesis of waterborne polyurethane; (e) the molecular structures of PEG and HDIt functional units, and the PH-BCPE with cross-linked 3D network structure by the copolymerization with *R* = 1.5 (ref. 31, copyrights 2002. Reproduced with permission from John Wiley and Sons. Ref. 32, copyrights 2002. Reproduced with permission from Elsevier. Ref. 33, copyrights 2002. Reproduced with permission from the American Chemical Society. Ref. 34, copyrights 2017. Reproduced with permission from Elsevier. Ref. 35, copyrights 2020. Reproduced with permission from Elsevier).

The polyether group is the most widely studied ‘soft segment’ matrix based on PU. The groups on the polyether molecule have a high donor number and high chain flexibility and are used to dissolve lithium salts and Li^+^.^7^ Subsequently, Zhu *et al.* synthesized PU-based electrolytes from a series of different polyether ‘soft segments’,^30^ including polydioxone (PDXL), polyethylene glycol (PEG), polytetrahydrofuran (PTMO), and PDXL/PEG [Fig. 3(a)]. They investigated the effects of different ‘soft segments’ on ionic conductivity. The results show that PDXL–PU exhibits lower *T*_g_ (−51.75 °C) due to the higher ether density of PXDL and higher ionic conductivity (2 × 10^−5^ S cm^−1^) by molecularly optimized PDXL-PU based SPE (LiClO_4_, [O]/[Li] = [12]). In addition, the ionic conductivity is also affected by the ratio of ‘soft-hard segment’. Wen *et al.* synthesized four polyether PUs composed of different ratios of 1, 4-butanediol (1, 4-bDO)/PEG [Fig. 3(b)].^36^ They found that the ionic conductivity decreased with increasing hard segment concentration. This may be ascribed to a reduction in the free volume, which reduces the mobility of the polymer chain. Hong *et al.* compared the ionic conductivity of linear polyurethanes (LPU) and hyperbranched chain polyurethanes (HPU) [Fig. 3(c)].^37^ The maximum ionic conductivity of the HPU-based SPE is 1.51 × 10^−5^ S cm^−1^ (25 °C). The practical application of polyether-based polyurethane in battery systems has also been reported. Cong *et al.* synthesized a water-based PU (WPU) from polyethylene glycol,^38^ diethylene glycol (DEG), dimethylene diisocyanate (HDI) and dimethylpropylene (DMPA) [Fig. 3(d)]. The developed WPU-based SPE (20 wt% LiTFSI in WPU) has an ionic conductivity of 7.3 × 10^−4^ S cm^−1^ at 60 °C. Subsequently, a new polyether polyester SPE (PH-BCPE) was designed by using a polyethylene glycol ‘soft segment’ and a hexamethylene diisocyanate trimer (HDIt) ‘hard segment’ [Fig. 3(e)].^39^ The optimized PH-BCPE SPE (*R*_(*n*PEG/*n*HDIt)_ = 1.87) has a high ionic conductivity (5.7 × 10^−4^ S cm^−1^) and a wide electrochemical window of up to 4.65 V at 55 °C.

**Fig. 4:**
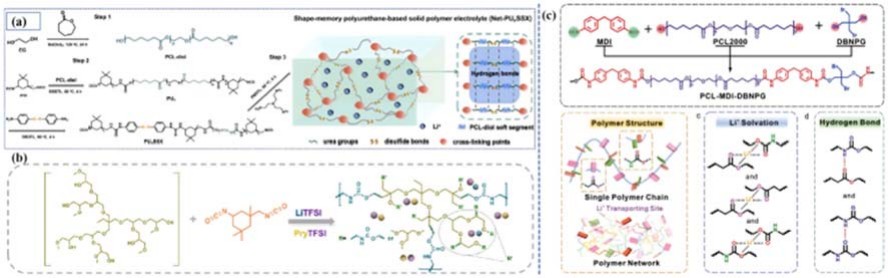
(a) Synthesis of Net-PUxSSX and the concept image showing the molecular structure; (b) schematic of HPU-based electrolyte; (c) schematic of PCL–MDI–DBNPG structure and schematic of the polymer structure, coordination effect of Li^+^ with O from polymer chains in SPE and hydrogen bond formed between “NH–” and “COO–” in SPE. (Reproduced with permission from Elsevier. Ref. 41, copyrights 2023. Reproduced with permission from Elsevier. Ref. 42, copyrights 2024. Reproduced with permission from John Wiley and Sons).

At present, the design of the molecular structure is the main method to synthesize PPES with specific functions. Wang and Xue *et al.* prepared a PU-based solid polymer electrolyte with shape memory properties through the molecular design of the ‘soft segment’ chains,^40^ which can control the temporary shape and restore the original shape with the help of the ‘soft segment’ in polycaprolactone (PCL) [Fig. 4(a)]. The abundant disulfide bond position and hydrogen bond in PUSPE also show the ability to heal easily under heat stimulation. The prepared flexible battery has inherent shape memory performance, which provides the possibility for matching with complex flexible devices. Wang *et al.* synthesized a novel hyperbranched polyurethane electrolyte (HPU1.5–IL1.5) by the reaction of HPEG (hyperbranched polyether) and IPDI (isopentanedione diisocyanate) in the presence of ionic liquid (IL) and LiTFSI [Fig. 4(b)].^43^ Owing to the structural advantages of hyperbranched polyurethane and the plasticizing effect of IL, the dissociation ability of lithium salt, the electrochemical stability of the electrolyte and the migration ability of Li^+^ are greatly enhanced. Furthermore, hydrogen bond interactions between the components can also enable microphase separation, thus helping to provide ion transport paths. In addition, the abundant hydrogen bonds in the electrolyte enhance the contact between the electrode and the electrolyte and promote the formation of a stable interface layer. This work provides a new idea for the design of advanced solid polymer electrolytes and also shows a good application prospect of hyperbranched polyurethane-based electrolytes in SLIBs. Huang synthesized a type of polyurethane-based polymer electrolyte with self-healing and flame-retardant properties that was obtained by designing the molecular structure of the polymer at the molecular level and selecting small molecular monomers with different functions through organic polymerization [Fig. 4(c)].^44^ The hydrogen bond between the ester group (–COO–) and the carbamate (–NH–COO–) gives the electrolyte good self-healing ability. The introduction of flame retardants also improves the mechanical strength of the polymer as a chain extender while participating in the polymerization reaction.

### 3.2 PPES/inorganic composite electrolytes

Among all the current types of solid electrolytes, inorganic electrolytes (oxides, sulfides, *etc.*) have ionic conductivity comparable to liquid electrolytes (10^−2^–10^−3^ S cm^−1^), but their poor flexibility becomes the most fatal defect of inorganic electrolytes. In addition, the high interface impedance generated by the “solid–solid” poor physical contact between the electrolyte and the electrode is also one of the disadvantages.^45^ The existence of high interface impedance reduces the capacity retention rate and efficiency of the battery at a high rate, which significantly influences the battery performance.^46,47^ In contrast, the solid polymer electrolyte (SPE) has the advantages of good flexibility and film formation,^19^ which can make up for the defect of inorganic solid electrolyte. Therefore, the preparation of polymer/ceramic composite electrolytes is an effective strategy to solve the shortcomings of inorganic ceramic electrolytes. Currently, physical blending is a simple and easy way to prepare polymer/inorganic electrolytes.^31^

**Fig. 5:**
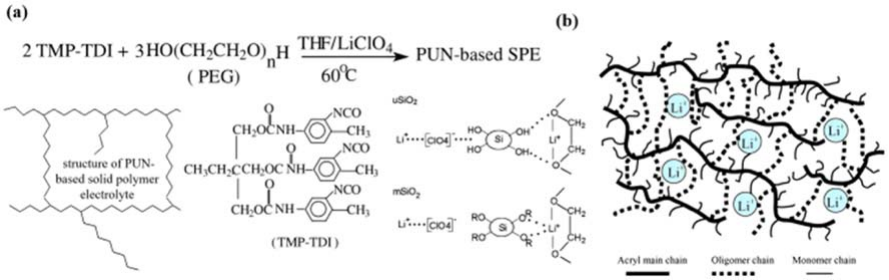
PPES/inorganic (inert fillers) composite electrolytes. (a) Preparation of PUN-based solid polymer electrolyte and pictorial model of the surface interaction between two forms of dispersed fumed silica filler and the PUN-LiClO_4_ electrolyte complex: uSiO_2_ with the native hydroxyl groups at the surface; mSiO_2_ with hydrophobic groups *R* at the surface. (b) Schematic of urethane acrylate polymer electrolyte (ref. 43, copyrights 2005. Reproduced with permission from Springer-Verlag. Ref. 44, copyrights 2004. Reproduced with permission from Elsevier).

For organic/inorganic composite electrolytes, inorganic fillers can be divided into inert fillers (such as SiO_2_, TiO_2_, and Al_2_O_3_.) and active fillers (such as Li_1.5_Al_0.5_Ge_1.5_(PO_4_)_3_ (LAGP),^32^ Li_1.3_Al_0.3_Ti_1.7_(PO_4_)_3_ (LATP), and Li_7_La_3_Zr_2_O_12_ (LLZO)). It is generally believed that the inert filler can reduce the crystallization of the polymer matrix, enhance the mechanical strength of the polymer and improve the ionic conductivity of the composite electrolyte. The active filler can directly supply Li^+^ to the system and increase the number of free lithium ions in the composite system. Fang introduced hydrophilic SiO_2_(uSiO_2_) and hydrophobic SiO_2_ (mSiO_2_) into polyether-PU network polymers (PUN) as typical inert fillers [Fig. 5(a)].^33^ It was found that when mSiO_2_ and uSiO_2_ reached about 1.67% and 1.25 wt%, respectively, the ionic conductivity increased by about 20 times, and the maximum ionic conductivity of SiO_2_/PUN-based composite SPE was 1.15 × 10^−5^ S cm^−1^ (mSiO_2_) and 1.02 × 10^−5^ S cm^−1^ (uSiO_2_) (30 °C). The increase in ionic conductivity of the system is attributed to the increase in the amorphous phase and Lewis acid–base interaction between uSiO_2_ and the polymer matrix and lithium salt. The amorphous phase promotes the movement of the polymer segment and thus the mobility of Li^+^. The interaction of uSiO_2_ with Lewis acid facilitates the dissociation of Li^+^, thereby increasing the carrier concentration. In another study by Jiang *et al.*,^34^ by introducing hydrophobic nano-SiO_2_ into PU-based SPEs, it was found that the ionic conductivity was approximately doubled compared to that of SPEs without nano-SiO_2_ [Fig. 5(b)]. Wu *et al.* prepared GPE films using PU,^35^ PVDF and 3 wt% TiO_2_ as substrates by electrospinning technology, the ionic conductivity of PU/PVDF/TiO_2_ composite GPE was 4.8 × 10^−3^ S cm^−1^ (25 °C), which is higher than that of PU/PVDF-based GPE at 3.2 × 10^−3^ S cm^−1^. Due to the inherent Li^+^ conductivity of active fillers, many scholars believe that active fillers can provide a highly effective way for Li^+^ transportation.^48^

**Fig. 6:**
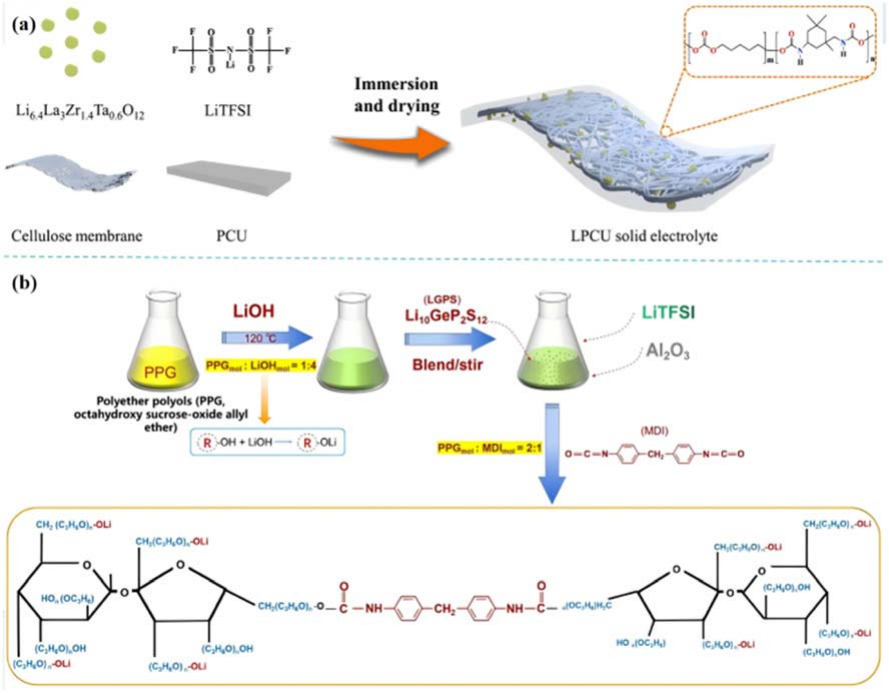
(a) Schematic of the preparation of LPCU electrolyte; (b) the preparation process of PLL [(PU–LGPS)/Li^+^] (ref. 47, copyrights 2023. Reproduced with permission from Elsevier. Ref. 12, copyrights 2022. Reproduced with permission from OAE Publishing Inc.).

Wang *et al.* have done a series of work in the field of PU-based solid polymer electrolyte,^41^ designed and optimized the rigid and flexible PU chain segment, and revealed its coordination with Li^+^ through theoretical calculation. When combined with Li_6.4_La_3_Zr_1.4_Ta_0.6_O_12_(LLZTO) [Fig. 6(a)], the ionic conductivity can reach 2.22 × 10^−4^ S cm^−1^ (25 °C), and the formation of lithium dendrites is effectively inhibited. Our group designed an LGPS–PU-based composite electrolyte by adding Li_10_GeP_2_S_12_(LGPS) particles into the ‘soft segment’ of PU^10^ and finally polymerized with diphenylmethane diisocyanate (MDI) to form a composite solid electrolyte [Fig. 6(b)]. The electrolyte has high ionic conductivity [3.1 × 10^−3^ S cm^−1^ (25 °C), 6.1 × 10^−3^ S cm^−1^ (80 °C)] and excellent Li^+^ transfer number (0.56), stable electrochemical window, and contributes to the construction of high-voltage all-solid lithium metal batteries. Despite the above-mentioned processes, there are few reports on active fillers reinforcing PU-based composite PE, and the above-mentioned Li^+^ pathway theory needs further study.

### 3.3 Effect of functional group modification on ionic conductivity of PPES

Although the organic/inorganic composite electrolyte improves the ionic conductivity of the system to a certain extent, the study of the intrinsic properties of polymer molecules is usually neglected. In contrast, molecular modification and functional group modification can effectively improve molecular intrinsic properties, so that high ionic conductivity can be achieved and the intrinsic advantages of polymers can be highlighted. Modifying the functional groups of the polymer molecules can effectively change the intrinsic properties of the molecules, and can improve the overall ionic conductivity effectively of the system.

First, compared with other polymers, PU has a unique structure (‘soft and hard segment’) and hydrogen bonds formed in the hard segment, giving PU and its derivatives self-healing properties and high adhesion properties.^13^ This not only ensures excellent electrochemical performance of PU but also enhances the compatibility between the electrolyte and electrode. Therefore, by introducing functional groups, a series of PU-based electrolytes with good ion transport performance and specific functions can be obtained. Among them, the “–NH” group in the hard segment of PU can form hydrogen bond interaction with “C=O” and “C–O–C” groups which yields PU self-healing properties.^42^ Wu *et al.* added amino terminal polyethylene glycol (NH_2_–PEG–NH_2_) to PU to enhance hydrogen bond interaction and prepared PU-based electrolyte with fast self-healing speed,^49^ and it has high *t*_Li^+^_ (0.44), low electronic conductivity (1.87 × 10^−8^ S cm^−1^), and high ionic conductivity (1.9 × 10^−4^ S cm^−1^).

**Fig. 7:**
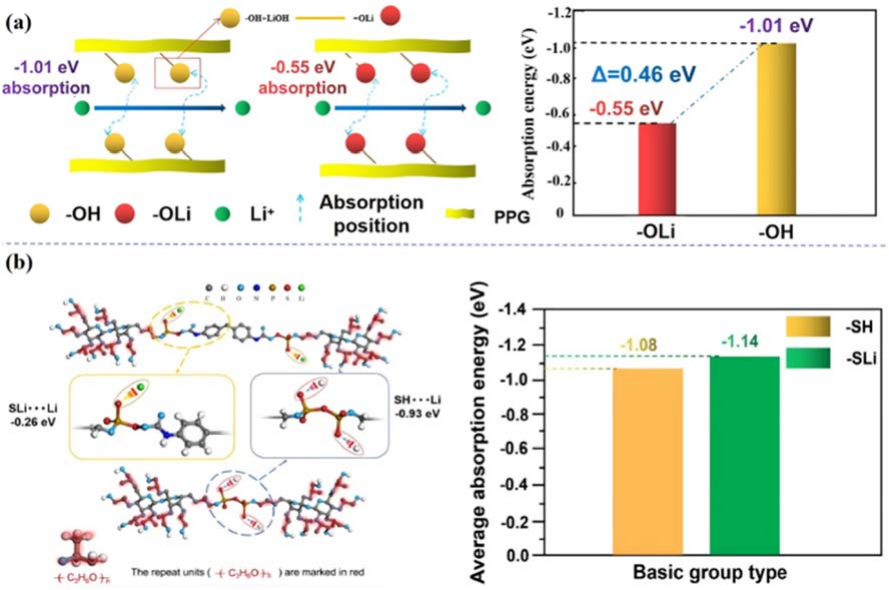
(a) Effect of the functional group “–OH” on the adsorption energy of ions in the system; (b) change of adsorption energy of Li^+^ by functional group modification (ref. 6 and 21, copyrights 2022. Reproduced with permission from the Royal Society of Chemistry).

Secondly, the functional group modification through the ‘soft segment’ chain part of the molecule can effectively reduce the kinetic properties of the functional group to Li^+^ in the system, which greatly increases the number of free Li^+^ in the system, and thus improves the ionic conductivity of the molecule. Our group modified the polar functional group “–OH” into “–OLi” through the reaction of “–OH” on the “soft segment” of the PU molecular chain with LiOH,^5^ which reduced the adsorption energy of the functional group successfully, and the adsorption energy is from “−1.01 eV” to “−0.55 eV”, increased the number of free Li^+^ in the system and improved the ionic conductivity of the system(2 × 10^−3^ S cm^−1^, 25 °C) [Fig. 7(a)]. On this basis, in our subsequent work,^17^ “–SLi” was introduced to replace “–SH” by reacting P_2_S_5_ with “–OH”, which further reduced the adsorption energy of the functional groups to ions, improved the affinity of the molecule to the electrode interface, and at the same time it had a high ionic conductivity (7.4 × 10^−4^ S cm^−1^ 25 °C, 4.3 × 10^−3^ S cm^−1^ ∼80 °C) [Fig. 7(b)].

### 3.4 Interfacial ion transport of PPES

**Fig. 8:**
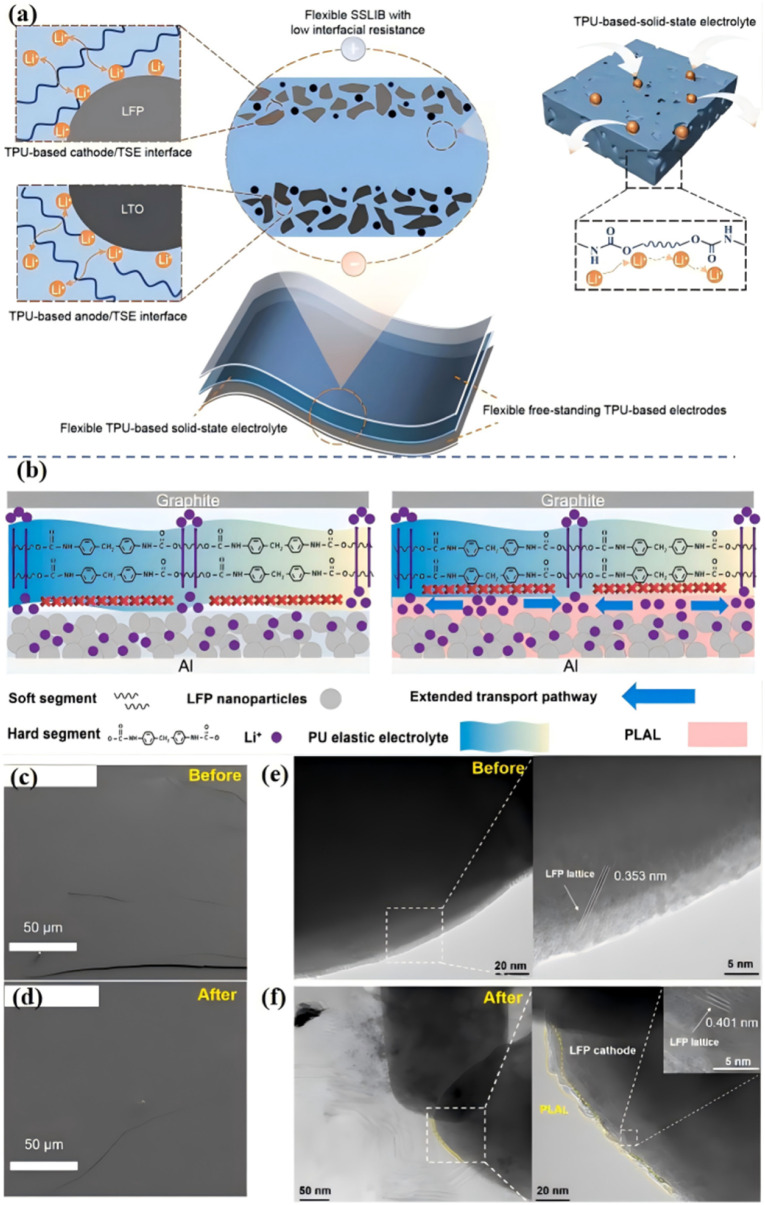
(a) Schematic of all-from-one flexible SSLIB consisting of TPU-based solid-state electrolyte and electrodes; (b) schematic of Li^+^ ion transport pathway on the contact interface between SPE and LFP with and without PLAL ISC and the diagram of the battery model and internal lithium-ion transition pathway of LFP|SPE|Graphite battery. The PLAL ISC significantly improves the ion transition kinetics and the ion-conducting channels are extended. Top view cryo-SEM images of PLAL ISC on the LFP cathode (c) before and (d) after 200 cycles; cryo-TEM images of the electrode materials (e) before and (f) after 200 cycles (the samples are treated by frozen slice); (ref. 52, copyrights 2024. Reproduced with permission from Springer-Verlag. Ref. 53 copyrights 2022. Reproduced with permission from Elsevier).

Low interface ion transport performance will lead to a high interface impedance, which will eventually lead to poor cyclic reversibility and rate performance, limiting the practical application of ASSLBs.^5,46,50^ Liang *et al.* proposed an all-homology strategy to construct a flexible solid-state lithium-ion battery with low interface impedance by using thermoplastic polyurethane as an ionic conductive framework for solid electrolytes and electrodes.^51^ Zou *et al.* achieved fast and stable Li^+^ transport by using a PU-based electrolyte (PO–PU–LiTFSI) with high ions conduction and high adhesion [Fig. 8(a)].^52^ The polar PU/urea group of the electrolyte reduces the jumping energy barrier of Li^+^, and achieves high ionic conductivity (1.8 × 10^−4^ S cm^−1^), high ions transfer number (0.54), and low activation energy (0.39 eV), thus achieving rapid Li^+^ transport. At the same time, due to the polarity of the PU group, the electrolyte has a high adhesion, ensuring the close contact of the interface and the self-healing electrode/electrolyte interface, thus ensuring the stable transport of Li^+^.

Although researchers have done a lot of work to improve the interface ion transport and reduce the interface impedance, it is still impossible to avoid the disadvantages of the special “soft–hard” segment structure of PU (hard segment does not conduct ions). At present, for the interface between the negative electrode and electrolyte, inorganic layers are mainly introduced to reduce the interface impedance.^54^ For the interface between the positive electrode and the electrolyte, the main method is to penetrate the positive electrode or introduce the organic/inorganic layer.^55^ Although these methods effectively enhance the stability of physical contact and battery cycle, the interface ion conductivity is low due to the poor trapping effect of the organic/inorganic layer on Li^+^, resulting in slow ion transport dynamics, which significantly affects the battery's magnification performance.^56^ Our group designed an organic PLAL ISC (PPG/Li^+^–Al_2_O_3_–LiOH, PLAL; interfacial superionic conductor, ISC) interface buffer layer based on the “soft segment” structure of PU polyether.^53^ The buffer with functional group modification using LiOH not only improves the ionic conductivity but also maintains the affinity of PLAL ISC (PPG/Li^+^–Al_2_O_3_–LiOH, PLAL; interfacial superionic conductor, ISC) to the positive electrode of LFP. As a coating layer for LFP, the buffer layer can overcome the shortcomings of a PU-based “hard segment” structure that does not conduct ions [Fig. 8(b)], expanding the ion transport path, and rebuilding the ion transport network. Therefore, it can be applied to high-performance ASSLBs with excellent rate performance and high capacity retention. The interface before and after the cycle was observed by cryo-SEM, indicating that the PLAL ISC can form a stable interface. This advantage can definitely improve the stability of the electrode at the high-rate cycling [Fig. 8(c and d)]. Cryo-TEM images of the internal structure of the cathode before and after cycling are shown in Fig. 8(e and f) to study the influence of PLAL ISC on the structure stability of LFP, which is closely related to the long cycle performance and capacity retention of assembled batteries. The PLAL ISC and electrode prepared directly permeate into each other to form not only a stable interface with the cathode but also an internal ion-conducting framework. The introduction of PLAL ISC can effectively construct an ion transition skeleton in the positive electrode material and accelerate the migration of Li^+^ from the positive LFP electrode to SPE. The assembled battery with PLAL ISC showed good cycle stability, with an ultra-high capacity retention rate of 92% after 1000 cycles at a rate of 10 C. The unique physical and chemical properties of the organic interface layer PLAL ISC, such as the electrowetting effect and interface adaptation, make it have an application potential in lithium-ion batteries, and the preparation method of this organic interface layer is also of great significance for the next generation of high-performance, low-cost energy storage devices.

## 4 Conclusions and perspectives

PU is a material with flexible design capability, easy molecular modifications (polymers, fillers and functional group modifications, *etc.*) and specific functions. Using these characteristics, a variety of specially designed PPES have been prepared and studied, which have high ionic conductivity, excellent mechanical strength, excellent thermal stability and excellent electrochemical properties. Therefore, they are considered highly promising candidates for high-performance ASSLBs. In this review, the main research progress of PPES is reviewed, and some molecular designs for improving ionic conductivity and interfacial ion transport are reviewed. However, despite the above developments, there are still many deficiencies and unsolved problems in practical applications. The challenges and prospects of PPES mainly focus on the following four aspects:

(1) Although PU-based gel states can accommodate these standards to some extent, they still contain liquid properties and fluidity, which poses potential safety concerns. In addition, the relatively low ionic conductivity obviously hinders the application of PU electrolytes in room-temperature ASSLBs. Therefore, PU-based SPEs with high room-temperature ionic conductivity (>10^−3^ S cm^−1^) should be explored. Some methods related to PU structural engineering can improve the ionic conductivity, such as introducing high conductivity segments to the polyether-based “soft segment” or designing polyether-based high free volume (combing, branching or hyperbranched) structures. At the same time, the composite PU-based SPE with a fast ionic conductor is also a practical method.

(2) More efforts are needed to explore the ion transport mechanism of PU-based SPE. Although great progress has been made in PU-based SPE, most of the mechanisms are based on the general profile of the polymer, not polyether-based PU individually, so the conduction mechanism is still unclear. For example, Li^+^ can only conduct electricity in the “soft segment” of PU/Li^+^, but whether hydrogen bonds and ‘hard segments’ will indirectly affect the transport of Li^+^ in the ‘soft segment’, there is no relevant report to explain it. Therefore, continued efforts are needed to uncover the ion conduction process in PU-based SPE. An in-depth understanding of the conductive mechanism is helpful for the development of high-performance polyether-based PU SPE.

(3) Polar functional groups can not only conduct Li^+^ but also have good compatibility with the electrode, such as “O=S=O”, “C=O”, “C≡N” and so on. By introducing these polar functional groups into PU, it is theoretically possible to obtain high-performance PU-based SPE. Secondly, by modifying the functional group, the adsorption performance of Li^+^ can be reduced, the number of free Li^+^ in the system can be greatly increased, and the ionic conductivity of the system can be improved. For example, sulfone or cyanide can enhance the high-pressure resistance of PU-based SPE, while maleic anhydride can improve interface compatibility.

(4) The ion-transport performance at the interface between the electrode and the electrolyte is a crucial step for the ASSLBs, therefore, the characterization method for its state is particularly important. Due to the limitation of existing characterization methods, the typical detection methods can not reflect the evolution of the electrode interface. In order to deeply understand and analyse the electrolyte/electrode interface of PU-based SPE, more novel and advanced technologies such as cryo-electron microscopy, surface-enhanced Raman spectroscopy (SERS), and synchrotron radiation are required.

We hope that all these aspects will have a guiding and positive effect on the further development of polyether-based polyurethane electrolytes to provide suitable materials for the upcoming ASSLBs.

## Data availability

All relevant data are within the paper.

## Author contributions

Peng Cui, Yifan Li and Chun Sun conceived the idea. Yifan Li wrote the manuscript, Yifan Li and Peng Cui contributed equally to this work. All authors have discussed the results, read the manuscript and agreed with its content. Peng Cui, Yifan Li, Chun Sun, Xingyu Tang and Siqi Zhang edited the manuscript. All authors discussed the results, read the manuscript and agreed with its content.

## Conflicts of interest

The authors declare that they have no known competing financial interests or personal relationships that could have appeared to influence the work reported in this paper.
